# Effect of metabolically healthy obesity on the development of arterial stiffness: a prospective cohort study

**DOI:** 10.1186/s12986-020-00474-8

**Published:** 2020-07-02

**Authors:** Yue Yuan, Jian-Jun Mu, Chao Chu, Wen-Ling Zheng, Yang Wang, Jia-Wen Hu, Qiong Ma, Yu Yan, Yue-Yuan Liao, Chen Chen

**Affiliations:** 1 Department of Cardiovascular Medicine, First Affiliated Hospital of Xi’an Jiaotong University, 277 Yanta West Street, Xi’an, 710061 China; 2 Key Laboratory of Molecular Cardiology of Shaanxi Province, Xi’an, China

**Keywords:** Metabolically healthy obesity, Arterial stiffness, Brachial-ankle pulse wave velocity

## Abstract

**Background:**

Metabolically healthy obesity (MHO) has been reported to be associated with the development of vascular damage by the carotid intima-media thickness, but the relationship between metabolic health and obesity phenotypes and arterial stiffness is still unknown. Our hypothesized that different metabolic health and obesity phenotypes might be associated with the development of arterial stiffness, and that subjects in MHO phenotype might not have increased risks of arterial stiffness compared with those in metabolically healthy nonobesity phenotype (MHNO), while metabolic unhealthy individuals might have increased risks of arterial stiffness.

**Methods:**

A prospective cohort of 2076 participants (aged 36–48 years) who were enrolled in the Hanzhong Adolescent Hypertension Cohort Study in 2017 was analyzed in a cross-sectional analysis. A subgroup of 202 participants from 2005 to 2017 was selected by an isometric sampling method and was included in the final longitudinal analysis.

**Results:**

We identified four metabolic health and obesity phenotypes for both the cross-sectional and longitudinal analyses as follows: MHNO, metabolically unhealthy nonobesity (MUNO), MHO, and metabolically unhealthy obesity (MUO). In the cross-sectional analysis, individuals with the MHO phenotype had the lowest brachial-ankle pulse wave velocity (baPWV) levels of the four phenotypes (*P* < 0.001), and participants with the MHO phenotype had a similar risk of arterial stiffness after fully adjustment [odds ratio (OR) = 0.99 (0.61–1.60)] as the MUNO subjects. Subjects with metabolically unhealthy status had a significantly higher risk of arterial stiffness than the MHNO individuals, particularly females (*P* < 0.005). In the longitudinal analysis, subjects with the MUNO and MUO phenotypes had a significantly higher risk of arterial stiffness than the MHNO individuals after adjustment for age and sex [OR = 5.21 (2.26–12.02), OR = 3.32 (1.18–9.32), respectively].

**Conclusions:**

The MHO phenotype did not significantly increase the progression of arterial stiffness. Metabolically unhealthy individuals (MUNO, MUO), regardless of obesity status, showed a worse effect for the development of arterial stiffness, particularly females.

**Trial registration:**

NCT02734472. Registered 12 April 2016 - Retrospectively registered, http:www.clinicaltrials.gov.

## Introduction

Obesity has become a nationwide public concern due to its high risk for the development of cardiovascular diseases (CVDs), including hypertension, diabetes mellitus and dyslipidemia [1–3]. It is worth mentioning that metabolic disturbances induced by obesity may not be present in all individuals with obesity. The metabolically healthy obesity (MHO) phenotype has been defined as individuals with obesity and an absence of cardiometabolic abnormalities (CAs) [4, 5]. Compelling evidence has demonstrated that compared with the “metabolically unhealthy obesity” (MUO) phenotype, the MHO phenotype has a lower risk of CVDs [6, 7]. However, several studies have claimed that the risk of all-cause and cardiovascular mortality in the MHO phenotype was similar to that in the MUO phenotype [8, 9]. The effect of metabolic health and obesity on the development of CVDs is controversial. The prevalence of MHO varies from 10 to 40% of individuals with obesity across studies based on different definitions [10].

Arterial stiffness is considered one of the earliest detectable measures of vascular damage, and many studies have indicated its predictive role in CVD [11, 12]. Recently, it has been reported that arterial stiffness is increased in overweight/obese subjects compared to controls with healthy body mass indexes (BMIs) [13, 14]. However, not all studies have reported greater arterial stiffness in subjects with obesity [15, 16]. Pulse wave velocity has been recognized as an objectively valid marker of arterial stiffness and is convenient and practical for use in the clinical investigation assessments [17]. The brachial-ankle pulse wave velocity (baPWV) is a reproducible index of elasticity and stiffness for both aortic and peripheral arteries and has emerged as a valuable predictor of cardiovascular mortality [18, 19]. Recent meta-analyses reported that baPWV could enhance the efficacy of prediction of the risk of development of CVD [20, 21]. Although previous studies have reported that metabolic complications are associated with increased arterial stiffness [22, 23], the relationship between arterial stiffness and the MHO phenotype had not been previously studied.

Our present study’s hypotheses were that (1) different metabolic health and obesity phenotypes might be associated with the development of arterial stiffness through the baPWV, and that (2) compared with those in metabolically healthy nonobesity (MHNO) phenotype, subjects in MHO phenotype might not have increased risks of arterial stiffness, while metabolic unhealthy individuals might have increased risks of arterial stiffness. Confirming this hypothesis have important implication regarding reduction of burden of arterial stiffness. We tested these hypotheses via cross-sectional and longitudinal analyses of a large prospective cohort study, comparing the effects of different metabolic health and obesity phenotypes on the development of arterial stiffness.

## Methods

### Study design

The Hanzhong Adolescent Hypertension Cohort Study is a prospective observational long-term follow-up study in northern China. We used the data of 4623 subjects (aged 6–18 years) who participated in the baseline health examinations between 1987 and 2017. Detailed cohort enrollment and information has been previously described [24].

**Fig. 1:**
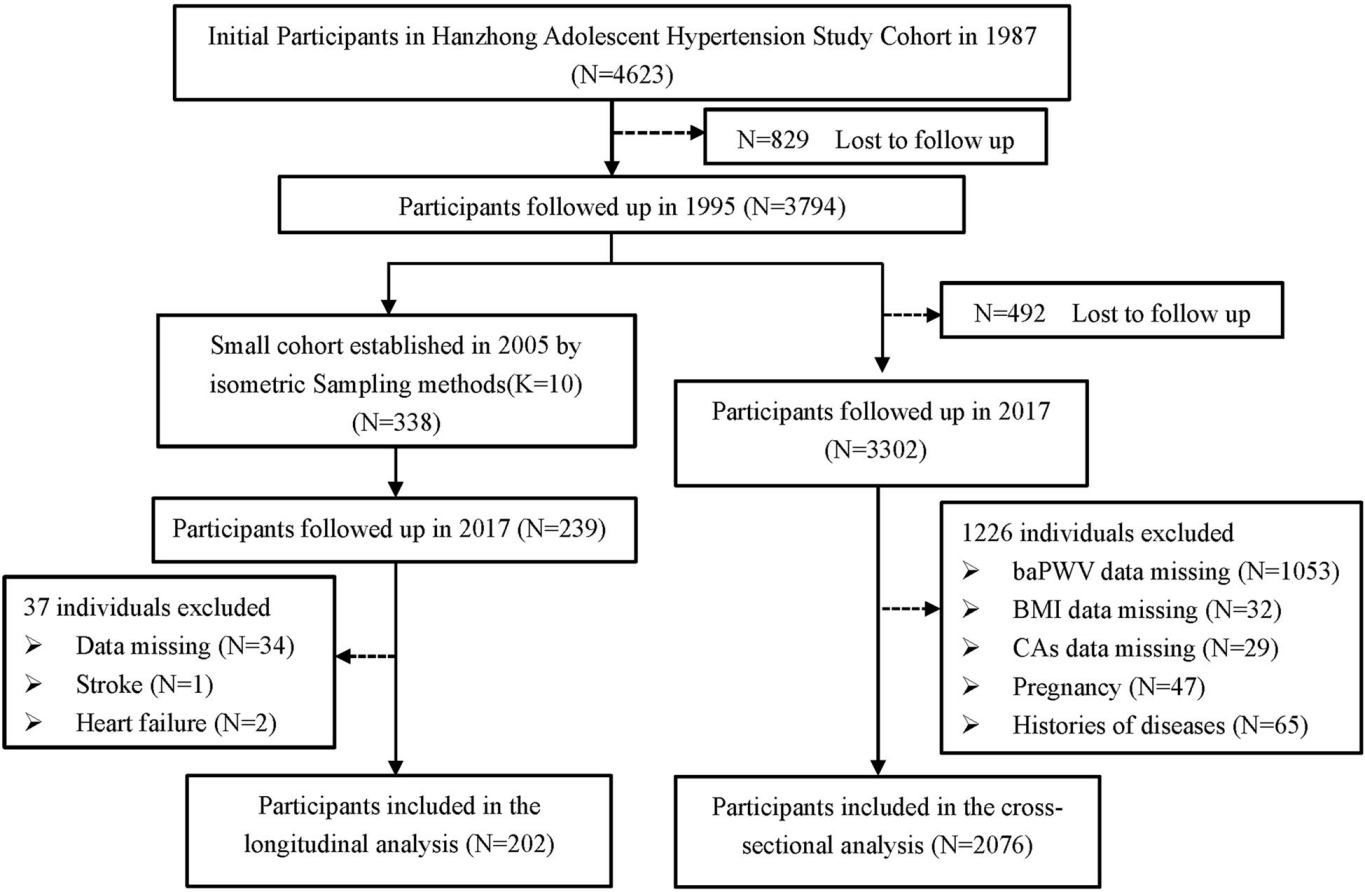
Flowchart of the study participants from Hanzhong Adolescent Hypertension cohort study (*n* = 2076). baPWV, brachial-ankle pulse wave velocity; BMI, body mass index; CA, cardiometabolic abnormality

The study consisted of two analyses: 1) A large-scale examination was conducted in the final follow-up year (2017), which included 3302 participants (aged 36–48 years). We excluded subjects who lacked baPWV data (*N* = 1053) and lacked BMI data (*N* = 32) and CAs (*N* = 29), including high blood pressure (BP), fasting plasma glucose (FPG) level, triglycerides (TG) and high-density lipoprotein cholesterol (HDL-C). Subjects with a history of stroke, heart failure, myocardial infarction, renal failure, infectious diseases (*N* = 65), and pregnancy (*N* = 47) were also excluded from this study. Finally, 2076 participants were included in the cross-sectional analysis. 2) We established a subgroup of 338 individuals from 2005 by an isometric sampling method and collected blood and urine samples. In the later follow-ups (2013 and 2017), 99 subjects were lost to follow-up, and the others were asked to participate in the sample collection. After excluding those with a history of stroke, heart failure, the absence of CAs, BMI and baPWV data (*N* = 37), 202 participants were included in the longitudinal analysis to address the relationship between the MHO phenotype and the development of baPWV. Figure 1 showed the selection of study population from Hanzhong Adolescent Hypertension cohort study.

This study was approved by the First Affiliated Hospital of Xi’an Jiao Tong University (trial registration number: NCT02734472; date of registration: 12/04/2016). The protocols were in accordance with the principles of the Helsinki Declaration. We obtained written informed consent from the children and their guardians in childhood and from the subjects in adulthood.

### Examination methods

A standardized self-questionnaire was used to collect the baseline data about clinical demographics, including histories of hypertension, diabetes mellitus, and hypertension, current smoking habits, current alcohol consumption and subsequent cardiovascular complications. Clinical characteristics, including physical examinations, were collected by trained investigators. BMI was calculated as body weight (kg) divided by the square of height (m^2^). BP was measured three times via an automated electronic device (OMRON Model HEM-725 FUZZY, Omron Company, Dalian, China) after at least 5 min of rest. The average of the three readings was calculated in the later analysis. We calculated the mean arterial pressure (MAP) by the formula MAP = 1/3 systolic blood pressure (SBP) + 2/3 diastolic blood pressure (DBP) (in mmHg). Biochemical assays of the fasting blood samples and first-void urine samples, which were collected by the experienced nurses, were conducted by an automatic biochemical analyzer (Hitachi, Ltd., Tokyo, Japan). The urinary microalbumin-to-creatinine ratio (uACR) was calculated by means of the following equation: urine microalbumin / urine creatinine. Estimated glomerular filtration rate (eGFR) was calculated using the formula adapted from the Modification of Diet in Renal Disease equation: eGFR = 175 × serum creatinine^-1.234^ × age^-0.179^ (× 0.79 for girls/women), where serum creatinine concentration is in milligrams per decilitre and age is in years. Trained staff measured the carotid intima-media thickness (cIMT) by high-resolution B-mode ultrasound in a double-blind manner. Subjects with SBP ≥ 140 mmHg or DBP ≥ 90 mmHg or who were receiving treatment for hypertension were classified as having hypertension. Subjects with FPG ≥ 7.0 mmol/L or treatment for diabetes were considered to have diabetes mellitus.

### Measurement of the baPWV and the definition of arterial stiffness

The assessment of baPWV was analyzed by a volume-plethysmographic apparatus (BP-203RPEII; Nihon Colin, Tokyo, Japan). Detailed collection information has been reported previously [25, 26]. Individuals were asked to maintain a supine position without a pillow. Trained investigators placed the ECG electrodes sandwiched between the two wrists and located the heart sound sensor at the left border of the sternum, with cuffs wrapped around both upper arms and ankles. Measurement of the path distance from the suprasternal notch to the brachium (Lb) or to the ankle on the same side (La) was obtained automatically, and baPWV was calculated by the formula baPWV (cm/s) = (La-Lb) / △T. The average of two measured values on the two sides was used to assess arterial stiffness. It has been acknowledged that a value of baPWV ≥1400 cm/s indicates arterial stiffness according to the Framingham Risk Score [26].

### Definitions of metabolic health and obesity

In this study, obesity was defined by a BMI of ≥25.0 kg/m^2^ based on the diagnostic criteria in Asian people, which are determined by the World Health Organization Western Pacific Region [27]. We used the National Cholesterol Education Program Adult Treatment Panel III (NCEP ATP III) criteria to define metabolic health, except for waist circumference (WC) due to its collinearity with BMI. In the NCEP ATP III criteria, those individuals with fewer than two of the following four CAs were considered metabolically healthy: elevated SBP/DBP of ≥135/80 mmHg or on antihypertensive treatment; high FPG of ≥100 mg/dL (5.6 mmol/L) or on hypoglycemic treatment; high TG of ≥1.7 mmol/L or on lipid-lowering therapy; low HDL-C (< 1.04 mmol/L in men and < 1.29 mmol/L in women) or on lipid-lowering medications [28]. Combined with the criteria for obesity and metabolic health, four phenotypes were defined to indicate different healthy statuses as follows: (1) metabolically healthy nonobesity (MHNO): BMI < 25 kg/m^2^ and < 2 CAs; (2) metabolically unhealthy nonobesity (MUNO): BMI < 25 kg/m^2^ and ≥ 2 CAs; (3) MHO: BMI ≥ 25 kg/m^2^ and < 2 CAs; (4) metabolically unhealthy obesity (MUO): BMI ≥ 25 kg/m^2^ and ≥ 2 CAs [29].

### Statistical analysis

All analyses were conducted in SPSS version 16.0 for Windows (SPSS Inc., Chicago, IL, USA). Baseline characteristics were presented according to the metabolic health and obesity phenotype. The continuous variables and categorical data are expressed as means ± standard deviations (SDs) and percentages, respectively. One-way ANOVA was used for three or more groups if the data met distribution and variance assumptions, while the Mann–Whitney U test was used for data that were not normally distributed. The qualitative variables were compared by using χ^2^ tests. Three models were established to adjust the odds ratios (ORs) and 95% confidence intervals (CIs) in the multivariate logistic regression model to determine the relationship between the metabolically healthy phenotypes and the development of arterial stiffness. Model 1 adjusted for sex and age; model 2 further adjusted for histories of hypertension, type 2 diabetes mellitus, hyperlipidemia, smoking habits and alcohol consumption; model 3 further adjusted for BMI, FPG, total cholesterol, TG, HDL-C, low-density lipoprotein cholesterol (LDL-C), uACR and eGFR. Statistical significance was set at a two-tailed *p* < 0.05.

## Results

### Clinical characteristics of the subjects included in the cross-sectional analysis

Four metabolic phenotypes were classified based on the definition of metabolic health and obesity as follows: the MHNO phenotype (*n* = 1187, 57.2%), the MUNO phenotype (*n* = 144, 6.9%), the MHO phenotype (*n* = 495, 23.8%) and the MUO phenotype (*n* = 250, 12.0%). Basic demographic characteristics and cardiometabolic profiles according to four metabolic phenotypes are presented in Table 1. Compared with subjects with the MHNO phenotype, those with the MHO phenotype had significantly higher levels of WC, SBP, DBP, FPG, TC, TG, LDL-C, serum urine acid, serum creatinine, uACRs, microalbumin and significantly lower levels of LDL-C and eGFR (all *P*s < 0.05). Compared with individuals with MUO, those with the MHO phenotype had significantly lower levels of WC, FPG, SBP, DBP, TC, TG, serum urine acid, serum creatinine, microalbumin, uACRs and significantly higher levels of HDL-C and eGFR (all *P*s < 0.05).

**Table 1:** Clinical characteristics of the study participants according to BMI and metabolic status in 2017

Characteristics	MHNO	MUNO	MHO	MUO	*P*
N	1187	144	495	250	–
Gender (M/F)	600/587	81/63	331/164	175/75	< 0.001
Age (years)	44.0 (41.0–46.0)	45.0 (42.0–46.0)	44.0 (41.0–46.0)	44.0 (41.0–46.0)	0.069
BMI (kg/m^2^)	22.4 (20.9–23.6)	23.5 (22.3–24.3)	26.6 (25.7–28.1)	27.4 (26.2–29.1)	< 0.001
Waist circumference (cm)	79.8 (75.2–84.2)	83.8 (78.9–87.3)	93.0 (88.9–96.9)	95.5 (90.9–100.1)	< 0.001
Current smoking (%)	450 (37.9)	64 (44.4)	70 (14.1)	77 (30.7)	< 0.001
Alcohol consumption (%)	294 (24.8)	42 (29.2)	16 (3.2)	18 (7.2)	< 0.001
Hypertension (n, %)	57 (4.8)	32 (22.2)	83 (16.8)	47 (18.7)	< 0.001
Diabetes mellitus (n, %)	12 (1.0)	15 (10.4)	254 (51.3)	135 (53.8)	< 0.001
Hyperlipidemia (n, %)	51 (4.3)	12 (8.3)	190 (38.4)	91 (36.3)	< 0.001
Heart rate (beats/min)	72.0 (66.0–80.0)	76.0 (69.0–85.0)	73.0 (67.0–78.0)	74.0 (68.0–80.3)	< 0.001
SBP (mmHg)	116.3 (108.9–124.3)	133.3 (129.7–139.3)	122.0 (115.7–129.0)	137.7 (131.7–150.0)	< 0.001
DBP (mmHg)	72.3 (66.3–79.0)	85.3 (79.3–90.0)	77.0 (71.7–83.3)	88.8 (84.0–95.7)	< 0.001
Fasting glucose (mmol/L)	4.48 (4.22–4.77)	4.76 (4.33–5.60)	4.62 (4.32–4.92)	4.85 (4.46–5.62)	< 0.001
Total cholesterol (mmol/L)	4.46 (4.00–4.94)	4.34 (4.00–4.90)	4.62 (4.11–5.23)	4.56 (4.14–5.05)	< 0.001
Triglycerides (mmol/L)	1.14 (0.85–1.55)	1.65 (1.15–2.36)	1.60 (1.12–2.21)	2.03 (1.59–2.90)	< 0.001
LDL- cholesterol (mmol/L)	2.46 (2.07–2.81)	2.52 (2.12–2.78)	2.60 (2.26–3.10)	2.56 (2.17–2.97)	< 0.001
HDL- cholesterol (mmol/L)	1.24 (1.08–1.43)	0.99 (0.90–1.13)	1.10 (1.00–1.24)	0.95 (0.86–1.03)	< 0.001
SUA (μmol/L)	263.9 (214.5–312.4)	283.1 (234.5–333.1)	304.5 (248.7–362.6)	322.1 (267.8–370.2)	< 0.001
Serum creatinine (μmol/L)	73.5 (65.3–83.9)	75.9 (66.4–86.8)	78.6 (69.0–87.4)	80.8 (71.3–90.2)	< 0.001
Urine albumin (mg/L)	6.30 (3.30–11.3)	9.10 (4.90–16.3)	8.15 (4.78–15.7)	11.9 (6.55–29.3)	< 0.001
uACR (mg/mmol)	0.87 (0.60–1.41)	1.18 (0.70–2.52)	1.04 (0.69–1.85)	1.57 (0.84–3.53)	< 0.001
eGFR (ml/min/1.73 m^2^)	99.07 (88.01–111.83)	94.71 (86.40–109.76)	96.22 (85.84–108.18)	93.96 (83.80–106.06)	< 0.001
cIMT (mm)	0.60 (0.50–0.72)	0.65 (0.55–0.75)	0.63 (0.55–0.75)	0.65 (0.55–0.75)	< 0.001
baPWV (cm)	1173.8 (1061.0–1297.8)	1366.8 (1257.3–1486.0)	1205.5 (1089.5–1366.5)	1402.3 (1265.8–1596.1)	< 0.001

*BMI* body mass index, *MHNO* metabolically healthy nonobesity, *MUNO* metabolically unhealthy nonobesity, *MHO* metabolically healthy obesity, *MUO* metabolically unhealthy obesity, *SBP* systolic blood pressure, *DBP* diastolic blood pressure, *LDL* low-density lipoprotein, *HDL* high-density lipoprotein, *SUA* serum uric acid, *uACR* urinary albumin-to-creatinine ratio, *eGFR* estimated glomerular filtration rate, *cIMT* carotid intima-media thickness, *baPWV* brachial-ankle pulse wave velocity. Non-normally distributed variables are expressed as the median (interquartile range). All other values are expressed as mean ± SD or n, %

### Association between metabolic obesity phenotypes and arterial stiffness

**Fig. 2:**
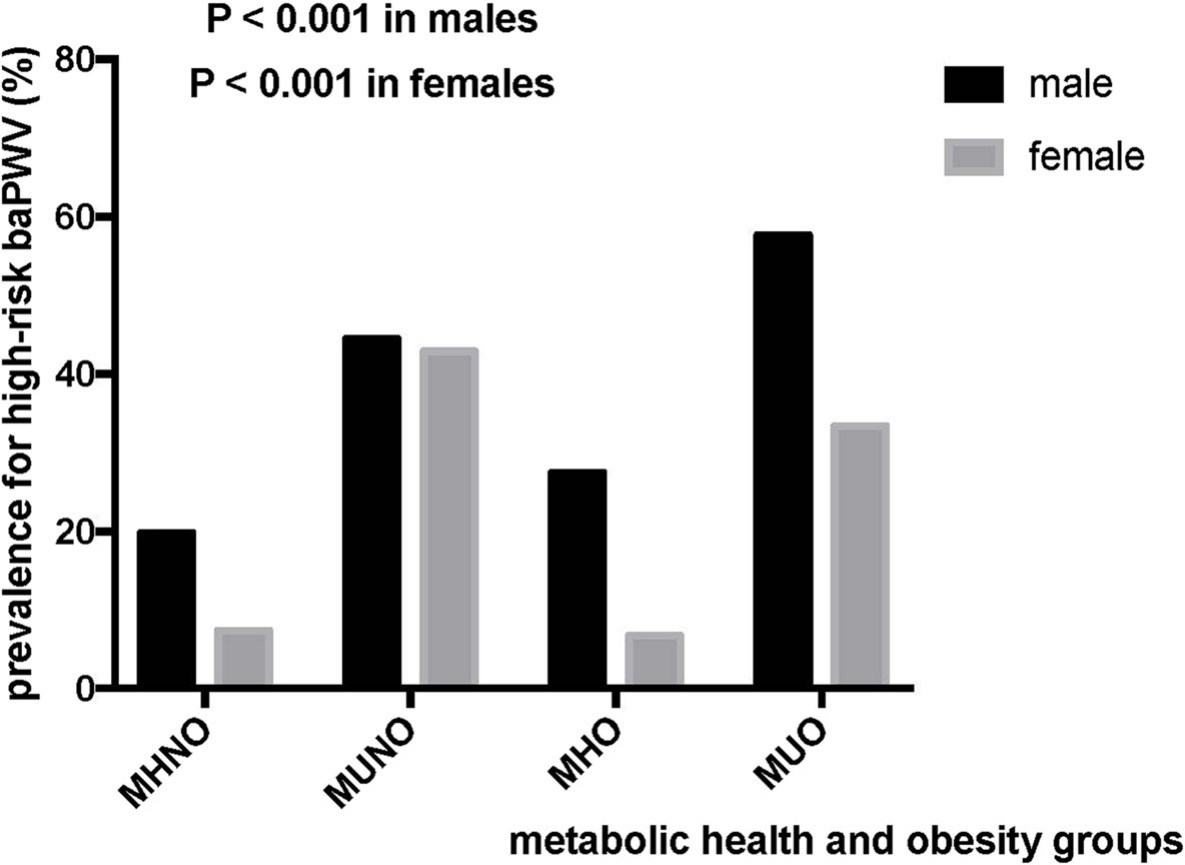
Prevalence of high-risk baPWV among the four metabolic health and obesity groups by sex (n = 2076). baPWV, brachial-ankle pulse wave velocity; MHNO: metabolically healthy nonobesity (males: *n* = 600; females: *n* = 587); MUNO: metabolically unhealthy nonobesity (males: *n* = 81; females: *n* = 63); MHO: metabolically healthy obesity (males: *n* = 331; females: *n* = 164); MUO: metabolically unhealthy obesity (males: *n* = 175; females: *n* = 75)

**Fig. 3:**
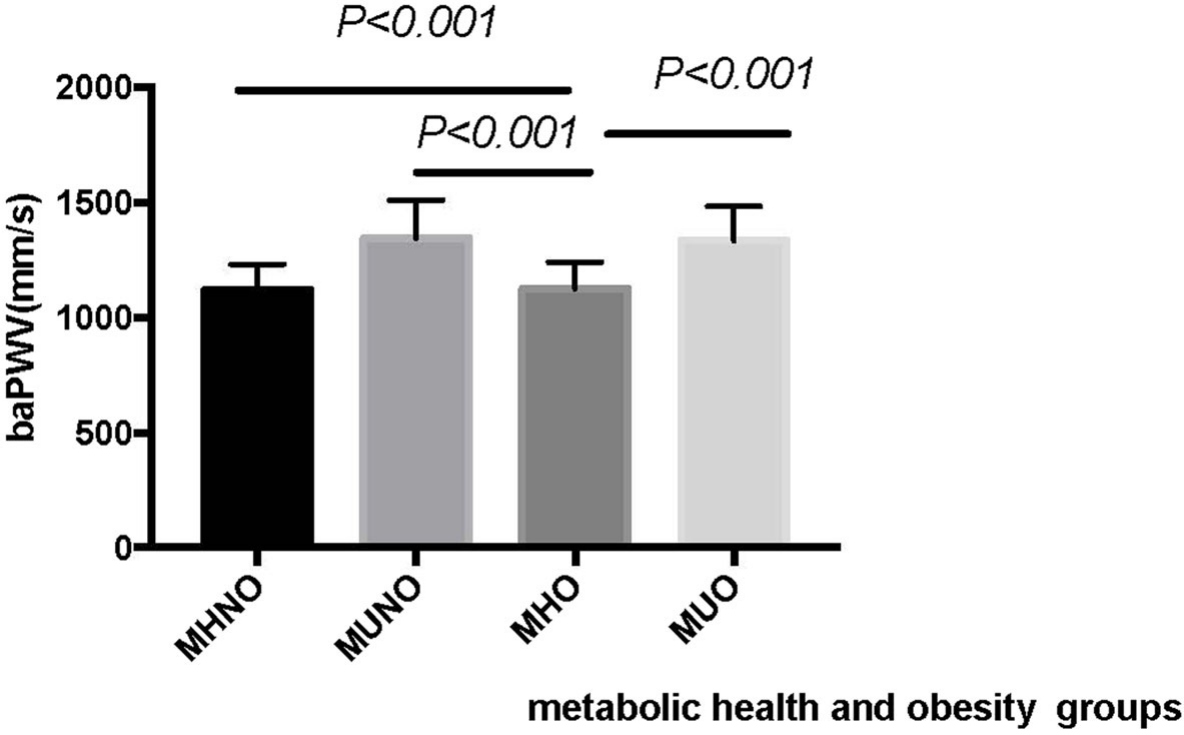
Comparison of the baPWV levels among the four metabolic health and obesity groups (n = 2076). baPWV, brachial-ankle pulse wave velocity; MHNO: metabolically healthy nonobesity (*n* = 1187); MUNO: metabolically unhealthy nonobesity (*n* = 144); MHO: metabolically healthy obesity (*n* = 495); MUO: metabolically unhealthy obesity(*n* = 250)

Figure 2 shows the prevalence of high-risk baPWV in the four phenotypes by sex. Males with the MUO phenotype had the highest prevalence (57.7%) of high-risk baPWV, while females with the MUNO phenotype had the highest prevalence (42.9%) of high-risk baPWV. Significant differences existed in both males and females among the four metabolic phenotypes. Among each of the four phenotypes, males had a higher prevalence of high-risk baPWV than females. A significant difference existed between the MHO phenotype and the other three phenotypes, as shown in the Fig. 3 (all *Ps* < 0.001).

Participants with unhealthy metabolic status (MUNO or MUO) had a significantly higher odds of arterial stiffness than those with the MHNO phenotype [OR = 5.05 (3.12–8.19) and OR = 4.56 (2.60–8.00), respectively], while those with the MHO phenotype had a similar odds [OR = 0.99(0.61–1.60)] of arterial stiffness in the fully adjusted model, as shown in the Table 2. We further analyzed the respective association between metabolic obesity phenotypes and incident arterial stiffness by sex (Additional file 1). As shown in Additional file 1, similar trends existed in both males and females subjects with MUNO, and individuals with the MUO phenotype had a significantly higher risk of arterial stiffness, which did not exist in those with the MHO phenotype. Additionally, compared with females, males had nearly 2 times the odds of high-risk baPWV in unhealthy metabolic status (MUNO, MUO) compared with males with the MHNO phenotype.

**Table 2:** Adjusted odds ratios and 95% confidence intervals of the association of metabolic health and obesity with high-risk baPWV in 2017 (*n* = 2076)

BMI and metabolic status	No. with high baPWV (%)	Unadjusted	Model 1	Model 2	Model 3
MHNO	162 (13.6)	1.00	1.00	1.00	1.00
MUNO	63 (43.8)	4.92^a^ (3.40–7.11)	4.87^a^ (3.33–7.12)	4.05^a^ (2.69–6.11)	5.05^a^(3.12–8.19)
MHO	102 (20.6)	1.64^a^ (1.25–2.16)	1.44^a^ (1.08–1.91)	1.19 (0.88–1.62)	0.99 (0.61–1.60)
MUO	126 (50.4)	6.43^a^ (4.77–8.66)	5.76^a^ (4.24–7.84)	4.49^a^ (3.21–6.29)	4.56^a^ (2.60–8.00)

baPWV, brachial-ankle pulse wave velocity; BMI, body mass index; MHNO, metabolically healthy nonobesity, MUNO, metabolically unhealthy nonobesity; MHO, metabolically healthy obesity; MUO, metabolically unhealthy obesity

MHNO was the reference group

Model 1: adjusted for age and sex;

Model 2: based on model 1 and history of hypertension, history of diabetes mellitus, history of hyperlipemia, smoke habits and alcohol consumption

Model 3: based on model 2 and further adjusted for BMI, fasting plasma glucose, total cholesterol, triglycerides, low-density lipoprotein cholesterol, high-density lipoprotein cholesterol, urinary albumin-to-creatinine ratio and estimated glomerular filtration rate in 2017

^a^significant values

### Distribution and demographic of subjects in the longitudinal analysis

Additional file 2 shows the demographics of the participants at baseline (2005) and the follow-up (2017). There were 114 males and 88 females in the longitudinal analysis. The median age of participants was 29.0 (27.0–32.0) years in 2005 and 41.0 (29.0–44.0) years in 2017. Compared with the baseline characteristics in 2005, more participants had a history of hypertension and diabetes mellitus in 2017 (*P* < 0.05). In addition, subjects had significantly higher levels of BMI, SBP, DBP, FPG, TC, TG, HDL-C and lower levels of LDL-C in 2017 compared with the initial examination (all *Ps* < 0.05). Overall, the MHO phenotype accounted for 10.2% (*n* = 23) of the total population (Table 3). At baseline, significant differences existed in the levels of BMI, BP, FPG, TC, TG, HDL-C and serum C-reactive protein among the four phenotypes (*P* < 0.05), while sex, heart rate, smoking habit, alcohol consumption, LDL-C, uACR and eGFR were not significantly different among the four groups (shown in Table 3).

**Table 3:** Baseline characteristics of the subjects according to metabolic obesity phenotypes in 2005 (*n* = 202)

Characteristics	MHNO	MUNO	MHO	MUO	*P*
N	105	51	23	23	–
Gender (M/F)	58/47	26/25	13/10	17/5	0.207
Age (years)	28.0 (27.0–32.0)	29.0 (26.0–32.0)	31.0 (29.0–33.0)	29.0 (27.0–31.0)	0.041
Current smoking (%)	33 (31.4)	17 (33.3)	12 (52.2)	10 (43.5)	0.232
Alcohol consumption (%)	33 (31.4)	19 (37.3)	10 (43.5)	12 (47.8)	0.402
Hypertension (n, %)	10 (9.5)	13 (25.5)	3 (13.0)	10 (43.5)	0.001
BMI (kg/m^2^)	21.4 (19.9–23.1)	22.1 (20.0–23.3)	25.6 (25.3–26.5)	27.5 (25.8–29.4)	< 0.001
Heart rate (beats/min)	72.0 (67.5–76.0)	74.0 (68.0–80.0)	78.0 (70.0–84.0)	70.0 (66.0–84.0)	0.147
SBP (mmHg)	117.1 ± 13.7	125.4 ± 21.1	117.4 ± 14.7	133.5 ± 15.3	< 0.001
DBP (mmHg)	74.2 ± 9.9	81.3 ± 14.0	77.1 ± 10.8	87.9 ± 8.9	< 0.001
Fasting glucose (mmol/L)	4.59 ± 0.56	4.85 ± 0.78	4.67 ± 0.46	5.39 ± 0.71	< 0.001
Total cholesterol (mmol/L)	4.41 ± 0.70	4.05 ± 0.80	4.61 ± 0.67	4.38 ± 0.61	0.014
Triglycerides (mmol/L)	1.10 (0.90–1.30)	1.50 (0.98–1.90)	1.30 (0.93–1.50)	1.90 (1.30–2.30)	< 0.001
LDL- cholesterol (mmol/L)	2.62 ± 0.47	2.44 ± 0.45	2.70 ± 0.39	2.64 ± 0.36	0.055
HDL- cholesterol (mmol/L)	1.12 ± 0.19	1.02 ± 0.18	1.16 ± 0.16	1.09 ± 0.15	0.010
SUA (μmol/L)	1.24 ± 0.27	1.19 ± 0.23	1.14 ± 0.18	1.04 ± 0.24	0.008
Serum creatinine (μmol/L)	2.55 (2.13–2.89)	2.55 (2.01–3.05)	2.58 (2.04–2.97)	2.42 (1.91–2.78)	0.313
Urine albumin (mg/L)	8.30 (4.55–13.65)	10.70 (4.90–24.80)	12.70 (5.80–38.90)	15.70 (6.60–29.90)	0.046
uACR (mg/mmol)	1.01 (0.66–1.84)	1.25 (0.65–3.14)	1.18 (0.78–3.73)	1.31 (0.58–9.03)	0.224
eGFR (ml/min/1.73 m^2^)	98.78 ± 17.80	105.02 ± 16.77	105.63 ± 23.03	104.36 ± 28.72	0.205
C-reactive protein (mg/L)	0.32 (0.18–0.68)	0.36 (0.16–0.85)	0.50 (0.29–0.90)	0.90 (0.38–2.04)	0.007

*BMI* body mass index, *MHNO* metabolically healthy nonobesity, *MUNO* metabolically unhealthy nonobesity, *MHO* metabolically healthy obesity, *MUO* metabolically unhealthy obesity, *SBP* systolic blood pressure, *DBP* diastolic blood pressure, *LDL* low-density lipoprotein, *HDL* high-density lipoprotein, *SUA* serum uric acid, *uACR* urinary albumin-to-creatinine ratio, *eGFR* estimated glomerular filtration rate. Non-normally distributed variables are expressed as the median (interquartile range). All other values are expressed as mean ± SD or n, %

### Prediction of arterial stiffness by metabolic obesity phenotypes

There were 55 (27.2%) participants with high-risk baPWV among the 202 participants in 2017. Participants with the MUNO phenotype had a significantly higher risk of arterial stiffness [OR = 5.21 (2.26–12.01), *P* < 0.001] after age and sex adjusted than those with the MHNO phenotype, and the trend consistently existed in the fully adjusted model (Table 4). Individuals with the MUO phenotype had the highest prevalence of arterial stiffness (43.5%) among the four phenotypes and had 3.32 times the risk of arterial stiffness after age- and sex-adjustment compared with individuals with the MHNO phenotype, but the trend was not significant in model 3. Individuals with the MHO phenotype had nearly 2 times the risk of arterial stiffness compared with those with the MHNO phenotype, but the OR was not significant (Table 4).

**Table 4:** Adjusted ORs of the association of metabolic health status with high-risk baPWV in 12-year follow-ups

BMI and metabolic status	No. with high baPWV (%)	Unadjusted OR (95%CI)	Model 1 OR (95%CI)	Model 2 OR (95%CI)	Model 3 OR (95%CI)
MHNO	16 (15.2)	1.00	1.00	1.00	1.00
MUNO	22 (43.1)	4.22^a^ (1.96–9.10)	5.21^a^ (2.26–12.01)	3.54^a^(1.42–8.82)	2.88^a^(1.09–7.59)
MHO	7 (30.4)	2.43 (0.86–6.85)	2.44 (0.79–7.52)	0.40 (0.08–1.92)	1.55 (0.30–8.06)
MUO	10 (43.5)	4.28^a^ (1.60–11.42)	3.32^a^ (1.18–9.32)	2.10 (0.68–6.54)	4.01 (0.75–21.56)

Odds ratio, OR; confidence interval, CI; baPWV, brachial-ankle pulse wave velocity; BMI, body mass index; MHNO, metabolically healthy nonobesity, MUNO, metabolically unhealthy nonobesity; MHO, metabolically healthy obesity; MUO, metabolically unhealthy obesity

MHNO was the reference group

Model 1: adjusted for age and sex;

Model 2: based on model 1 and history of hypertension, history of diabetes mellitus, history of hyperlipemia, smoke habits and alcohol consumption

Model 3: based on model 2 and further adjusted for BMI, fasting plasma glucose, total cholesterol, triglycerides, low-density lipoprotein cholesterol, high-density lipoprotein cholesterol, urinary albumin-to-creatinine ratio and estimated glomerular filtration rate in 2017

^a^significant values

## Discussion

With this prospective cohort study, we were the first to compare the risk of arterial stiffness by metabolic health and obesity phenotypes. We confirmed the two hypotheses that metabolic health and obesity phenotypes was associated with the development of arterial stiffness, and unhealthy individuals with either obesity or nonobesity status had a significantly higher risk of arterial stiffness than individuals with the MUNO phenotype, which did not exist in those with the MHO phenotype.

The increase in the PWV with age in adulthood was reported to be not only due to the decrease in elasticity caused by the degeneration of the arterial wall but also the consequence of many metabolic abnormalities such as obesity and CAs [30]. Recent studies have demonstrated that the baPWV is associated with metabolic syndrome and increases with increasing numbers of metabolic syndrome components [23, 31] . It is worth mentioning that different from those studies, we classified metabolic health and obesity status and use BMI to reflect obesity instead of WC. In this study, individuals with the MHO phenotype showed a similar risk of arterial stiffness compared with the MHUO phenotype in the cross-sectional analysis. Additionally, at the 12-year follow-up, individuals with the MHO phenotype showed 2.44 times the age- and sex-adjusted risks of arterial stiffness compared with those without unhealthy and obesity statuses, although this difference was not statistically significant. Our results acknowledge the harmful effect of obesity and unhealthy metabolic status and highlight that metabolic health status may be the main determinants of the high-risk baPWV.

In Asian population studies, females have been reported to show greater association with arterial stiffness than males as the number of metabolic components increases [32, 33], which was similar to our findings. In contrast to our findings, Angelo Scuteri et al. reported that the impact of metabolic abnormalities on the development of arterial stiffness was similar in men and women [34], while another study showed that the baPWV in males increased as the number of components increased [31]. Age and race differences in the study population, the statistical methods, and the pulse wave velocity measurements may all have contributed to this discrepancy.

The prevalence of MHO ranges from 6 to 75% based on several sociodemographic factors, such as sex, age, and race, and the prevalence of MHO seems to be higher in the Asia population compared with Caucasian or multiethnic populations [35]. In recent years, the particular effect of MHO phenotype on cardiovascular risk events is still controversial. Compelling evidence has found no excess risk of CVD among MHO subjects compared to those with the MUNO phenotype [36], while other studies have demonstrated that there is no healthy pattern of increased weight [8]. N Miyai et al. reported that none of the adiposity profiles was selected as an independent determinant of the baPWV in a stepwise multivariate regression model, which suggested that the association of obesity with baPWV is mainly mediated by the presence of elevated BP, dyslipidemia and impaired glucose tolerance [37]. By contrast, some studies have claimed that excess body fat is identified as an independent risk factor for accelerated arterial stiffening [38, 39]. In this study, our findings showed that despite these controversies, there is no doubt that particular attention should be given to metabolically unhealthy subjects within the nonobese population for the risks of arterial stiffness.

The mechanism of the relationship between metabolic health and obesity phenotypes and the development of arterial stiffness has not been fully elucidated. It has been reported that extracellular matrix remodeling, perivascular adipose tissue inflammation, and immune cell dysfunction contribute to the development of arterial stiffness in individuals with obesity [40]. Arterial stiffness and accelerated vascular aging were reported to be related to cellular growth, oxidative stress and vascular inflammation caused by excessive stimulation of angiotensin type 1 receptors, as well as mineralocorticoid receptors [41]. Several possible mechanisms may explain the phenomenon that a higher risk of arterial stiffness was not evident in individuals with the MHO phenotype. Preserved insulin sensitivity, a specific fat distribution with low visceral and ectopic fat accumulation compared with subcutaneous fat depots may play crucial roles in the pathogenesis of the abovementioned mechanisms, as well as the complex interconnection among genetic, environmental, and behavioral factors [28, 42].

The main limitations of this study were the large loss to follow-up and small samples of participants in the longitudinal analysis. Racial homogeneity was also a limitation of this study. The absence of data on dietary intake and physical activity was another limitation. BMI does not provide information on the distribution of body fat. Moreover, A minimal resting time of 25 min is needed before measuring stabilized blood pressure in subjects addressed for vascular investigations [43]. 5-min rest before clinic BP evaluation may affect the precision and accuracy of the measurement. The clinical value of our findings for obese subjects of different races is limited since the cutoff for obesity in America is BMI of ≥30.0 kg/m^2^ [44, 45]. The criteria of obesity in this study leads to the concern that application of the current BMI cut-off points will overestimate obesity-related risks in America. Multiple and larger-scale cohort studies are needed to validate our findings. Despite these limitations, this study was the first to determine the predictive value of incident arterial stiffness by metabolic health and obesity phenotypes. A combination of cross-sectional and longitudinal analyses indicated the comprehensive results.

## Conclusion

Our results elucidate the effect of vascular stiffness on metabolic health and obesity status by showing that unhealthy individuals with either obesity or nonobesity showed a significantly higher risk of arterial stiffness, particularly in females. Individuals with the MHO phenotype showed no significant harmful effect of high-risk baPWV compared with metabolically healthy and nonobese subjects. Our findings suggest that the interventions for unhealthy metabolic conditions are necessary to prevent the development of arterial stiffness, irrespective of obesity status, which has important meanings for the efficient allocation of resources to the target treatment.

## Supplementary information

**Additional file 1 **Adjusted odds ratios and 95% confidence intervals of the association of metabolic health and obesity with high baPWV by sex in 2017 (*n* = 2076).

**Additional file 2 **Characteristics of the study participants at baseline and during follow-ups (*n* = 202).

## Data Availability

All data generated or analyzed during this study are included in this published article [and its supplementary information files].

## Abbreviations

MHO: Metabolically healthy obesity
MHNO: Metabolically healthy nonobesity
MUNO: Metabolically unhealthy nonobesity
MUO: Metabolically unhealthy obesity
baPWV: Brachial-ankle pulse wave velocity
OR: Odds ratio
CVD: Cardiovascular disease
CA: Cardiometabolic abnormality
BMI: Body mass index
BP: Blood pressure
FPG: Fasting plasma glucose
TG: Triglycerides
HDL-C: High-density lipoprotein cholesterol
MAP: Mean arterial pressure
SBP: Systolic blood pressure
DBP: Diastolic blood pressure
uACR: Urinary microalbumin-to-creatinine ratio
eGFR: Estimated glomerular filtration rate
cIMT: carotid intima-media thickness
WC: Waist circumference
SD: Standard deviation
CI: Confidence interval
LDL-C: Low-density lipoprotein cholesterol
