# Supplementary material for: Effect of metabolically healthy obesity on the development of arterial stiffness: a prospective cohort study
Source: Nutr Metab (Lond). 2020 Jul 2;17:50. doi: 10.1186/s12986-020-00474-8 (PMC7330959; doi:10.1186/s12986-020-00474-8)
Supplement: Supplementary file 1 — Additional file 1 Adjusted odds ratios and 95% confidence intervals of the association of metabolic health and obesity with high baPWV by sex in 2017 (n = 2076). [file 12986_2020_474_MOESM1_ESM.docx]

| Adjusted odds ratios and 95% confidence intervals of the association of metabolic health and obesity with high baPWV by sex in 2017 (n= 2076) | | | | | |
| --- | --- | --- | --- | --- | --- |
| BMI and metabolic status | No. with high baPWV (%) | Unadjusted | Model 1 | Model 2 | Model 3 |
| Male | 347(29.2) |  |  |  |  |
| MHNO | 119(19.8) | 1.00 | 1.00 | 1.00 | 1.00 |
| MUNO | 36(44.4) | 3.23* (2.00-5.24) | 3.18* (1.96-5.17) | 2.84* (1.69-4.78) | 3.91* (2.13-7.18) |
| MHO | 91(27.5) | 1.53* (1.12-2.10) | 1.61* (1.17-2.21) | 1.31 (0.93-1.84) | 1.09 (0.64-1.85) |
| MUO | 101(57.7) | 5.52* (3.85-7.91) | 5.57* (3.88-8.01) | 4.16* (2.87-6.32) | 3.76* (1.98-7.16) |
| Female | 106(11.9) |  |  |  |  |
| MHNO | 43(19.3) | 1.00 | 1.00 | 1.00 | 1.00 |
| MUNO | 27(42.9) | 9.49* (5.27-17.08) | 9.31* (5.15-16.86) | 7.25* (3.64-14.44) | 9.59* (3.94-23.36) |
| MHO | 11(49.6) | 0.91 (0.46-1.81) | 0.90 (0.45-1.80) | 0.89 (0.43-1.86) | 0.79 (0.24-2.62) |
| MUO | 25(33.3) | 6.33* (3.57-11.21) | 6.17* (3.47-10.99) | 5.43* (2.85-10.38) | 6.64* (1.92-22.97) |
| baPWV, brachial-ankle pulse wave velocity; BMI, body mass index; MHNO, metabolically healthy nonobesity, MUNO, metabolically unhealthy nonobesity; MHO, metabolically healthy obesity; MUO, metabolically unhealthy obesity.  MHNO was the reference group.  Model 1: adjusted for age in 2017;  Model 2: based on model 1 and history of hypertension, history of diabetes mellitus, history of hyperlipemia, smoke habits and alcohol consumption in 2017.  Model 3: based on model 2 and further adjusted for BMI, fasting plasma glucose, total cholesterol, triglycerides, low-density lipoprotein cholesterol, high-density lipoprotein cholesterol, urinary albumin-to-creatinine ratio and estimated glomerular filtration rate in 2017.  *significant values | | | | | |
