# Supplementary material for: Effect of metabolically healthy obesity on the development of arterial stiffness: a prospective cohort study
Source: Nutr Metab (Lond). 2020 Jul 2;17:50. doi: 10.1186/s12986-020-00474-8 (PMC7330959; doi:10.1186/s12986-020-00474-8)
Supplement: Supplementary file 2 — Additional file 2 Characteristics of the study participants at baseline and during follow-ups (n = 202). [file 12986_2020_474_MOESM2_ESM.doc]

Characteristics of the study participants at baseline and during follow-ups (n= 202)

| Characteristics | Baseline in 2005 | Follow-up in 2017 | *P* for trend |
| --- | --- | --- | --- |
| Gender (M/F) | 114/88 | 114/88 | − |
| Age (years) | 29.0(27.0-32.0) | 41.0 (39.0-44.0) | <0.001 |
| Current smoking (%) | 72 (35.6) | 80 (39.6) | 0.411 |
| Alcohol consumption (%) | 73 (36.1) | 51 (25.2) | 0.018 |
| Hypertension (n, %) | 36 (17.8) | 58 (28.7) | 0.010 |
| Diabetes mellitus (n, %) | 1 (0.5) | 15 (7.4) | <0.001 |
| Body mass index (kg/m^2^) | 22.9±3.5 | 24.6±3.5 | <0.001 |
| Heart rate (beats/min) | 73.0 (68.0-78.0) | 74.0 (66.0-80.0) | 0.920 |
| SBP (mmHg) | 121.7±14.8 | 124.7±16.3 | <0.001 |
| DBP (mmHg) | 78.3 ±10.6 | 78.4±11.1 | <0.001 |
| Fasting glucose (mmol/L) | 4.75 ± 0.67 | 4.69 ± 0.90 | 0.001 |
| Total cholesterol (mmol/L) | 4.36±0.69 | 4.57±0.79 | <0.001 |
| Triglycerides (mmol/L) | 1.20 (0.95-1.63) | 1.36 (0.94-1.93) | 0.005 |
| LDL- cholesterol (mmol/L) | 2.59±0.44 | 2.53±0.68 | <0.001 |
| HDL- cholesterol (mmol/L) | 1.10 (0.98-1.20) | 1.17 (1.02-1.36) | <0.001 |
| SUA (μmol/L) | − | 279.7 (232.4-353.4) | − |
| Serum creatinine (μmol/L) | − | 76.1±14.1 | − |
| uACR(mg/mmol) | − | 1.17 (0.68-2.57) | − |
| eGFR(ml/min/1.73 m^2^) |  | 99.70(88.22-112.31) |  |
| cIMT(mm) | − | 0.64 (0.55-0.75) | − |
| baPWV(cm) | − | 1285.5 (1171.5-1411.0) | − |

SBP, systolic blood pressure; DBP, diastolic blood pressure; LDL, low-density lipoprotein; HDL, high-density lipoprotein; SUA, serum uric acid; uACR, urinary albumin-to-creatinine ratio; eGFR, estimated glomerular filtration rate; cIMT, carotid Intima-Media Thickness; baPWV, brachial-ankle pulse wave velocity. Non-normally distributed variables are expressed as the median (interquartile range). All other values are expressed as mean ± SD or n, %.
